# Enumeration of the Multiplicative Degree-Kirchhoff Index in the Random Polygonal Chains

**DOI:** 10.3390/molecules27175669

**Published:** 2022-09-02

**Authors:** Wanlin Zhu, Xianya Geng

**Affiliations:** School of Mathematics and Big Data, Anhui University of Science & Technology, Huainan 232000, China

**Keywords:** multiplicative degree-Kirchhoff index, random polygonal chains, expected value, extremal value, average value

## Abstract

Multiplicative degree-Kirchhoff index is a very interesting topological index. In this article, we compute analytical expression for the expected value of the Multiplicative degree-Kirchhoff index in a random polygonal. Based on the result above, we also get the Multiplicative degree-Kirchhoff index of all polygonal chains with extremal values and average values.

## 1. Introduction

In this paper, we only consider simple and finite connected graphs. The topological index is a mathematical descriptor of molecular structure, which is obtained by performing some numerical operation on the matrix representing the molecular graph. It is an invariant graph, directly generated from the molecular structure, and used to reflect the size, shape, branch and other structural features of the molecule, so as to realize the numerical value of molecular structure information. Molecular topological indices are widely used because of their simplicity, objectivity and freedom from experience and experiment. More than 200 different types of topological indices have been proposed.

It is well known that the emergence and development of graph theory is closely related to the study of chemical molecular graphs. Using topological indices, physicochemical properties and activity parameters of compounds to construct QSPR/QSAR models and to evaluate and predict their properties has become one of the most active fields in chemistry research. In recent years, increasing attention has been paid to the development of chemical workers. A graphical representation of a compound contains atoms as nodes and bonds as edges. For more detailed information, we can refer to [1,2,3,4].

Topological indices can be used to describe chemical structures and are related to the physical properties, thermodynamic parameters, chemical properties, biological activities and carcinogenicity of compounds. A graph is an ordered two-tuples G=(V(G),E(G)), where V(G) is a nonempty set and E(G) is a set disjoint from V(G). V(G) and E(G) are the vertex set and edge set of *G*. The number of edges incident at *v* in *G* is called the degree of the vertex *v* in *G* and is denoted by $d_{G}\left(v\right)$. If *u* and *v* are in the same component of *G*, we define d(u,v) to be the length of a shortest u−v path in *G* [5,6,7].

In 1993, Klein and Randi$\overset{´}{c}$, academicians of the International Academy of Mathematical Chemists, discussed the application of effective resistance in chemistry and named the effective resistance of a graph as the resistance distance of a graph. This was the first time the concept of resistance distance was put forward in the world, and its proposers pointed out that the resistance distance has more advantages than the shortest distance in the study of molecular communication distance and other aspects [8].

The Kirchhoff index is defined as

(1) $$Kf\left(G\right)=\sum_{\left\{x,y\right\}\subseteq V_{G}}r\left(x,y\right).$$

In 2007, the multiplicative degree-Kirchhoff index was defined as [9]

(2) $$Kf^{*}\left(G\right)=\sum_{\left\{x,y\right\}\subseteq V_{G}}d\left(x\right)d\left(y\right)r\left(x,y\right).$$

**Figure 1:**
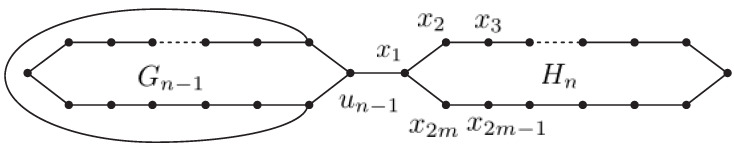
A polygonal chain $G_{n}$ with *n* polygons.

**Figure 2:**
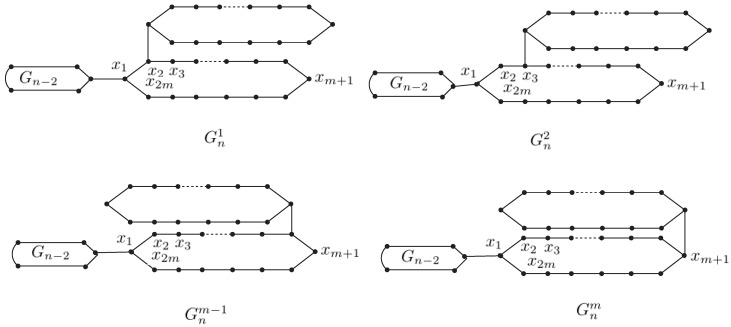
*m* types of local arrangements in a polygonal chain.

A random polygonal chain $G_{n}$ with *n* polygons is composed of a polygonal chain $G_{n-1}$ with n−1 polygons, where a new terminal polygon $H_{n}$ is adjacent to a cut edge, see Figure 1. For n≥3, the terminal polygon $H_{n}$ can be connected in *m* ways, which can describe as a permutation of $G_{n}^{1}$,$G_{n}^{2}$,$G_{n}^{3}$,…,$G_{n}^{m}$. see Figure 2. A random polygon chain $G_{n}\left(p_{1},p_{2},p_{3},\ldots ,p_{m-1}\right)$ is the polygonal chain obtained by successively adding terminal polygons. In each addition m(=3,4,…,n) can randomly select one of the *m* connection modes:

- $G_{m-1}\to G_{2m}^{1}$ with probability $p_{1}$,
- $G_{m-1}\to G_{2m}^{2}$ with probability $p_{2}$,
- $G_{m-1}\to G_{2m}^{3}$ with probability $p_{3}$,
- ⋮          ⋮                          ⋮
- $G_{m-1}\to G_{2m}^{m-1}$ with probability $p_{m-1}$,
- $G_{m-1}\to G_{2m}^{m}$ with probability $p_{m}=1-p_{1}-p_{2}-p_{3}-\cdots -p_{m-1}$,

where the probabilities $p_{1}$, $p_{2}$, $p_{3}$, …, $p_{m-1}$ are constants, independent of the parameter *m*.

Let $G_{n}$ be a polygonal chain with *n* polygons $H_{1}$, $H_{2}$, …, $H_{n}$. $u_{t}\omega_{t}$ links $H_{t}$ and $H_{t+1}$ with $u_{t}\in V_{H_{t}}$ in $G_{n}$, $\omega_{t}\in V_{H_{t+1}}$ for t=1,2,⋯,n−1. Evidently, both $\omega_{t}$ and $u_{t+1}$ are the vertices in $H_{t+1}$ and $d\left(\omega_{t},u_{t+1}\right)\in \left\{1,2,3,\ldots ,n\right\}$. In particular, $G_{n}$ is the meta-chain $M_{n}\left(p_{m}=1\right)$, the ortho-chain $O_{n}^{1}$,$O_{n}^{2}$, …, $O_{n}^{m-2}\left(p_{i+1}=1,1\leq i\leq m-2\right)$ and the para-chain $L_{n}\left(p_{m}=1\right)$ if $d(\omega_{t},u_{t+1})=1$ (i.e., $p_{1}=1$), $d(\omega_{t},u_{t+1})$ = 2 (i.e., $p_{2}=1$), $d(\omega_{t},u_{t+1})$ = 3 (i.e., $p_{3}=1$), …, $d(\omega_{t},u_{t+1})$= m(i.e., $p_{m}=1$) for all t∈{1,2,…,n−2}, respectively.

Huang, Kuang and Deng [10] calculated the random polyphenyl and spiro chains, while Zhang and Li et al. [11] calculated the random polyphenylene chain expected values of the multiplicative Kirchhoff index. For more information, we can refer to [12,13,14,15,16,17,18,19,20,21,22,23,24,25,26,27,28,29,30]. We compute analytical expression for the expected value of the multiplicative degree-Kirchhoff index in a random polygonal. We also obtain the multiplicative degree-Kirchhoff index with extremal values and average values of all polygonal chains. These results will play a positive role in the study of chemical and physical properties of compounds, drug design and environmental pollution prediction [31,32].

## 2. The Multiplicative Degree-Kirchhoff Index in a Random Polygonal Chain

In this part, in random polygonal chain we will compute the expected value of Multiplicative degree-Kirchhoff index. For a random polygonal chain $G_{n}$, the Multiplicative degree-Kirchhoff index is a random variable. Actually, $G_{n+1}$ is $G_{n}$ connected by an edge to a new terminal polygonal $H_{n+1}$, here $H_{n+1}$ is composed of vertices $x_{1}$, $x_{2}$, $x_{3}$,…,$x_{2m}$, and the new edge is $u_{n}x_{1}$; see Figure 1. For all $v\in V_{G_{n}}$,

(3) $$\begin{array}{cc} \; & r\left(x_{1},v\right)=r\left(u_{n},v\right)+1,\\ \; & r\left(x_{2},v\right)=r\left(u_{n},v\right)+1+\frac{1\cdot (2m-1)}{1+(2m-1)}=r\left(u_{n},v\right)+1+\frac{2m-1}{2m},\\ \; & r\left(x_{3},v\right)=r\left(u_{n},v\right)+1+\frac{2\cdot (2m-2)}{2+(2m-2)}=r\left(u_{n},v\right)+1+\frac{4m-4}{2m},\\ \; & \;\;\;\;\;\;\;\;\;\;\;\;\;\vdots \;\;\;\;\;\;\;\;\;\;\;\;\;\;\;\;\vdots \;\;\;\;\;\;\;\;\;\;\;\;\;\;\;\;\vdots \;\;\;\;\;\\ \; & r\left(x_{m},v\right)=r\left(u_{n},v\right)+1+\frac{\left(m-1)\cdot (m+1\right)}{\left(m-1)+(m+1\right)}=r\left(u_{n},v\right)+1+\frac{m^{2}-1}{2m},\\ \; & r\left(x_{m+1},v\right)=r\left(u_{n},v\right)+1+\frac{m\cdot m}{m+m}=r\left(u_{n},v\right)+1+\frac{m^{2}}{2m},\\ \; & r\left(x_{m+2},v\right)=r\left(u_{n},v\right)+1+\frac{\left(m+1)\cdot (m-1\right)}{\left(m+1)+(m-1\right)}=r\left(u_{n},v\right)+1+\frac{m^{2}-1}{2m},\\ \; & \;\;\;\;\;\;\;\;\;\;\;\;\;\vdots \;\;\;\;\;\;\;\;\;\;\;\;\;\;\;\;\vdots \;\;\;\;\;\;\;\;\;\;\;\;\;\;\;\;\vdots \;\;\;\;\;\\ \; & r\left(x_{2m-1},v\right)=r\left(u_{n},v\right)+1+\frac{(2m-2)\cdot 2}{(2m-2)+2}=r\left(u_{n},v\right)+1+\frac{4m-4}{2m},\\ \; & r\left(x_{2m},v\right)=r\left(u_{n},v\right)+1+\frac{(2m-1)\cdot 1}{(2m-1)+1}=r\left(u_{n},v\right)+1+\frac{2m-1}{2m}. \end{array}$$

(4) $$\sum_{v\in V_{G_{n}}}d_{G_{n+1}}\left(v\right)=\left[\left(2m-2\right)\cdot 2+2\cdot 3\right]n-1=\left(4m+2\right)n-1.$$

And,

(5) $$\begin{array}{cc} \; & \sum_{i=1}^{2m}d\left(x_{i}\right)r\left(x_{1},x_{i}\right)=\frac{4m^{2}-1}{3}=\frac{8m^{3}-2m}{6m},\\ \; & \sum_{i=1}^{2m}d\left(x_{i}\right)r\left(x_{2},x_{i}\right)=\frac{4m^{2}-1}{3}+\frac{1\cdot (2m-1)}{2m}=\frac{8m^{3}+4m-3}{6m},\\ \; & \sum_{i=1}^{2m}d\left(x_{i}\right)r\left(x_{3},x_{i}\right)=\frac{4m^{2}-1}{3}+\frac{2\cdot (2m-2)}{2m}=\frac{8m^{3}+10m-12}{6m},\\ \; & \;\;\;\;\;\;\;\;\;\;\;\;\;\;\;\vdots \;\;\;\;\;\;\;\;\;\;\;\;\;\;\;\;\vdots \;\;\;\;\;\;\;\;\;\;\;\;\;\;\;\;\vdots \;\;\;\;\;\\ \; & \sum_{i=1}^{2m}d\left(x_{i}\right)r\left(x_{m},x_{i}\right)=\frac{4m^{2}-1}{3}+\frac{\left(m-1)\cdot (m+1\right)}{2m}=\frac{8m^{3}+3m^{2}-2m-3}{6m},\\ \; & \sum_{i=1}^{2m}d\left(x_{i}\right)r\left(x_{m+1},x_{i}\right)=\frac{4m^{2}-1}{3}+\frac{m\cdot m}{2m}=\frac{8m^{2}+3m-2}{6m},\\ \; & \sum_{i=1}^{2m}d\left(x_{i}\right)r\left(x_{m+2},x_{i}\right)=\frac{4m^{2}-1}{3}+\frac{\left(m+1)\cdot (m-1\right)}{2m}=\frac{8m^{3}+3m^{2}-2m-3}{6m},\\ \; & \;\;\;\;\;\;\;\;\;\;\;\;\;\;\;\vdots \;\;\;\;\;\;\;\;\;\;\;\;\;\;\;\;\vdots \;\;\;\;\;\;\;\;\;\;\;\;\;\;\;\;\vdots \;\;\;\;\;\\ \; & \sum_{i=1}^{2m}d\left(x_{i}\right)r\left(x_{2m-1},x_{i}\right)=\frac{4m^{2}-1}{3}+\frac{(2m-2)\cdot 2}{2m}=\frac{8m^{3}+10m-12}{6m},\\ \; & \sum_{i=1}^{2m}d\left(x_{i}\right)r\left(x_{2m},x_{i}\right)=\frac{4m^{2}-1}{3}+\frac{(2m-1)\cdot 1}{2m}=\frac{8m^{3}+4m-3}{6m}. \end{array}$$

**Theorem** **1.** The $E(Kf^{*}\left(G_{n}\right))$(n≥1) of the random polygonal chain $G_{n}$ is

$$\begin{array}{cc} E(Kf^{*}\left(G_{n}\right) & )=\left\{\left(4m^{3}+12m^{2}+9m+2\right)-\left(2m+1\right)\sum_{i=1}^{m-1}\left[\left(2m^{2}+5m+2\right)-\frac{2m+i(2m-i)}{m}\left(2m+1\right)\right]p_{i}\right\}\frac{n^{3}}{3}\\ \; & +\left\{\frac{4m^{3}-4m^{2}-19m-8}{3}+\left(2m+1\right)\sum_{i=1}^{m-1}\left[\left(2m^{2}+5m+2\right)-\frac{2m+i(2m-i)}{m}\left(2m+1\right)\right]p_{i}\right\}n^{2}\\ \; & -\left\{\left(8m^{2}-8m-9\right)+2\left(2m+1\right)\sum_{i=1}^{m-1}\left[\left(2m^{2}+5m+2\right)-\frac{2m+i(2m-i)}{m}\left(2m+1\right)\right]p_{i}\right\}\frac{n}{3}-1. \end{array}$$

**Proof.**  The random polygonal chain $G_{n+1}$ is obtained by successively adding a new terminal polygon $H_{n+1}$ to $G_{n}$ by an edge, here $H_{n+1}$ is composed of vertices $x_{1}$, $x_{2}$, $x_{3}$,…, $x_{2m}$, and the new edge is $u_{n}x_{1}$; see Figure 1. Through (2), one has

$$\begin{array}{cc} Kf^{*}\left(G_{n+1}\right)\; & =\sum_{\left\{u,v\right\}\subseteq V_{G_{n}}}d\left(u\right)d\left(v\right)r\left(u,v\right)+\sum_{v\in V_{G_{n}}}\sum_{x_{i}\in V_{H_{n+1}}}d\left(v\right)d\left(x_{i}\right)r\left(v,x_{i}\right)+\sum_{\left\{x_{i}x_{j}\right\}\subseteq V_{H_{n+1}}}d\left(x_{i}\right)d\left(x_{j}\right)r\left(x_{i},x_{j}\right). \end{array}$$

Note that

$$\begin{array}{cc} \sum_{\left\{u,v\right\}\subseteq V_{G_{n}}}d\left(u\right)d\left(v\right)r\left(u,v\right) & =\sum_{\left\{u,v\right\}\subseteq V_{G_{n}}\setminus \left\{u_{n}\right\}}d\left(u\right)d\left(v\right)r\left(u,v\right)+\sum_{v\in V_{G_{n}}\setminus \left\{u_{n}\right\}}d_{G_{n+1}}\left(u_{n}\right)d\left(v\right)r\left(u_{n},v\right)\\ & =\sum_{\left\{u,v\right\}\subseteq V_{G_{n}}\setminus \left\{u_{n}\right\}}d\left(u\right)d\left(v\right)r\left(u,v\right)+\sum_{v\in V_{G_{n}}\setminus \left\{u_{n}\right\}}\left(d_{G_{n}}\left(u_{n}\right)+1\right)d\left(v\right)r\left(u_{n},v\right)\\ & =Kf^{*}\left(G_{n}\right)+\sum_{v\in V_{G_{n}}}d\left(v\right)r\left(u_{n},v\right). \end{array}$$

□

Recall that $d(x_{1})=3$ and $d(x_{i})=2$ for i∈{2,3,4,…,2m}. From (3) and (4), We have

$$\begin{array}{cc} \; & \sum_{v\in V_{G_{n}}}\sum_{x_{i}\in V_{H_{n+1}}}d\left(v\right)d\left(x_{i}\right)r\left(v,x_{i}\right)\\ = & \sum_{v\in V_{G_{n}}}d\left(v\right)[3\left(r\left(u_{n},v\right)+1\right)+2\left(r\left(u_{n},v\right)+1+\frac{1\cdot (2m-1)}{2m}\right)\\ \; & +2\left(r\left(u_{n},v\right)+1+\frac{2\cdot (2m-2)}{2m}\right)+2\left(r\left(u_{n},v\right)+1+\frac{3\cdot (2m-3)}{2m}\right)\\ \; & +\cdots +2\left(r\left(u_{n},v\right)+1+\frac{\left(m-1)\cdot (m+1\right)}{2m}\right)+2\left(r\left(u_{n},v\right)+1+\frac{m\cdot m}{2m}\right)\\ \; & +2\left(r\left(u_{n},v\right)+1+\frac{\left(m+1)\cdot (m-1\right)}{2m}\right)+\cdots +2\left(r\left(u_{n},v\right)+1+\frac{(2m-2)\cdot 2}{2m}\right)\\ \; & +2(r\left(u_{n},v\right)+1+\frac{(2m-1)\cdot 1}{2m})]\\ = & \sum_{v\in V_{G_{n}}}d\left(v\right)\left[\left(4m+1\right)r\left(u_{n},v\right)+\frac{4m^{2}+12m+2}{3}\right]\\ = & \left(4m+1\right)\sum_{v\in V_{G_{n}}}d\left(v\right)r\left(u_{n},v\right)+\frac{4m^{2}+12m+2}{3}\left[\left(4m+2\right)n-1\right]. \end{array}$$

From (5), one has,

$$\begin{array}{cc} \; & \sum_{\left\{x_{i}x_{j}\right\}\subseteq V_{H_{n+1}}}d\left(x_{i}\right)d\left(x_{j}\right)r\left(x_{i},x_{j}\right)=\frac{1}{2}\sum_{i=1}^{2m}d\left(x_{i}\right)\left(\sum_{j=1}^{2m}d\left(x_{j}\right)r\left(x_{i},x_{j}\right)\right)\\ = & \frac{1}{2}[3\times \frac{4m^{2}-1}{3}+2\times \left(\frac{4m^{2}-1}{3}+\frac{1\cdot (2m-1)}{2m}\right)+2\times \left(\frac{4m^{2}-1}{3}+\frac{2\cdot (2m-2)}{2m}\right)\\ & +\cdots +2\times \left(\frac{4m^{2}-1}{3}+\frac{\left(m-1)\cdot (m+1\right)}{2m}\right)+2\times \left(\frac{4m^{2}-1}{3}+\frac{m\cdot m}{2m}\right)\\ \; & +2\times \left(\frac{4m^{2}-1}{3}+\frac{\left(m+1)\cdot (m-1\right)}{2m}\right)+\cdots +2\times \left(\frac{4m^{2}-1}{3}+\frac{(2m-1)\cdot 1}{2m}\right)]\\ = & \frac{8m^{3}+4m^{2}-2m-1}{3}. \end{array}$$

Then

(6) $$\begin{array}{c} \begin{array}{cc} Kf^{*}\left(G_{n+1}\right)= & Kf^{*}\left(G_{n}\right)+\left(4m+2\right)\sum_{v\in V_{G_{n}}}d\left(v\right)r\left(u_{n},v\right)+\frac{4m^{2}+12m+2}{3}\left[\left(4m+2\right)n-1\right]\\ + & \frac{8m^{3}+4m^{2}-2m-1}{3}. \end{array} \end{array}$$

For a random polygonal chain $G_{n}$, the expected value of the number $\sum_{v\in V_{G_{n}}}d\left(v\right)r\left(u_{n},v\right)$ is a random variable. We can represent it

$$R_{n}:=E\left(\sum_{v\in V_{G_{n}}}d\left(v\right)r\left(u_{n},v\right)\right).$$

Substituting $R_{n}$ into (6), we can get the recurrence formula of $E(Kf^{*}\left(G_{n}\right))$

$$\begin{array}{cc} E(Kf^{*}\left(G_{n+1}\right))= & E\left(Kf^{*}\left(G_{n}\right)\right)+\left(4m+2\right)R_{n}+\frac{16m^{3}+56m^{2}+32m+4}{3}n+\frac{8m^{3}-14m-3}{3}. \end{array}$$

We continue to consider the following *m* possibilities.

**Way 1.**$G_{n}⟶G_{n+1}^{1}$. In this way, $u_{n}$ gives the same result with the vertex $x_{2}$ or $x_{2m}$. Then, $\sum_{v\in V_{G_{n}}}d\left(v\right)r\left(u_{n},v\right)$ is described as $\sum_{v\in V_{G_{n}}}d\left(v\right)r\left(x_{2},v\right)$ or $\sum_{v\in V_{G_{n}}}r\left(v\right)d\left(x_{2m},v\right)$ with probability $p_{1}$.
**Way 2.**$G_{n}⟶G_{n+1}^{2}$. In this way, $u_{n}$ gives the same result with the vertex $x_{3}$ or $x_{2m-1}$. Then, $\sum_{v\in V_{G_{n}}}d\left(v\right)r\left(u_{n},v\right)$ is described as $\sum_{v\in V_{G_{n}}}d\left(v\right)r\left(x_{3},v\right)$ or $\sum_{v\in V_{G_{n}}}d\left(v\right)r\left(x_{2m-1},v\right)$ with probability $p_{2}$.
**Way 3.**$G_{n}⟶G_{n+1}^{3}$. In this way, $u_{n}$ gives the same result with the vertex $x_{4}$ or $x_{2m-2}$. Then, $\sum_{v\in V_{G_{n}}}d\left(v\right)r\left(u_{n},v\right)$ is described as $\sum_{v\in V_{G_{n}}}d\left(v\right)r\left(x_{4},v\right)$ or $\sum_{v\in V_{G_{n}}}d\left(v\right)r\left(x_{2m-2},v\right)$ with probability $p_{3}$.
⋮      ⋮                        ⋮
**Way m-3.**$G_{n}⟶G_{n+1}^{m-3}$. In this way, $u_{n}$ gives the same result with the vertex $x_{m-2}$ or $x_{m+4}$. Then, $\sum_{v\in V_{G_{n}}}d\left(v\right)r\left(u_{n},v\right)$ is described as $\sum_{v\in V_{G_{n}}}d\left(v\right)r\left(x_{m-2},v\right)$ or $\sum_{v\in V_{G_{n}}}d\left(v\right)r\left(x_{m+4},v\right)$ with probability $p_{m-3}$.
**Way m-2.**$G_{n}⟶G_{n+1}^{m-2}$. In this way, $u_{n}$ gives the same result with the vertex $x_{m-1}$ or $x_{m+3}$. Then, $\sum_{v\in V_{G_{n}}}d\left(v\right)r\left(u_{n},v\right)$ is described as $\sum_{v\in V_{G_{n}}}d\left(v\right)r\left(x_{m-1},v\right)$ or $\sum_{v\in V_{G_{n}}}d\left(v\right)r\left(x_{m+3},v\right)$ with probability $p_{m-2}$.
**Way m-1.**$G_{n}⟶G_{n+1}^{m-1}$. In this way, $u_{n}$ gives the same result with the vertex $x_{m}$ or $x_{m+2}$. Then, $\sum_{v\in V_{G_{n}}}d\left(v\right)r\left(u_{n},v\right)$ is described as $\sum_{v\in V_{G_{n}}}d\left(v\right)r\left(x_{m},v\right)$ or $\sum_{v\in V_{G_{n}}}d\left(v\right)r\left(x_{m+2},v\right)$ with probability $p_{m-1}$.
**Way m.**$G_{n}⟶G_{n+1}^{m}$, then $u_{n}$ is the vertex $x_{m+1}$. Then, $\sum_{v\in V_{G_{n}}}d\left(v\right)r\left(u_{n},v\right)$ is described as $\sum_{v\in V_{G_{n}}}d\left(v\right)r\left(x_{m+1},v\right)$ with probability $1-p_{1}-p_{2}-p_{3}-\ldots -p_{m-3}-p_{m-2}-p_{m-1}$.

According to the above discussion, we obtain

$$\begin{array}{cc} R_{n}= & p_{1}\sum_{v\in V_{G_{n}}}d\left(v\right)r\left(x_{2},v\right)+p_{2}\sum_{v\in V_{G_{n}}}d\left(v\right)r\left(x_{3},v\right)+p_{3}\sum_{v\in V_{G_{n}}}d\left(v\right)r\left(x_{4},v\right)\\ & +\cdots +p_{m-3}\sum_{v\in V_{G_{n}}}d\left(v\right)r\left(x_{m-2},v\right)+p_{m-2}\sum_{v\in V_{G_{n}}}d\left(v\right)r\left(x_{m-1},v\right)+p_{m-1}\sum_{v\in V_{G_{n}}}d\left(v\right)r\left(x_{m},v\right)\\ & +\left(1-p_{1}-p_{2}-p_{3}-\ldots -P_{m-3}-P_{m-2}-p_{m-1}\right)\sum_{v\in V_{G_{n}}}d\left(v\right)r\left(x_{m+1},v\right)\\ = & p_{1}\left[\sum_{v\in V_{G_{n-1}}}d\left(v\right)r\left(u_{n-1},v\right)+\left(1+\frac{1\cdot (2m-1)}{2m}\right)\left(\left(4m+2\right)n-1\right)+\left(\frac{4m^{2}-1}{3}+\frac{1\cdot (2m-1)}{2m}\right)\right]\\ \; & +p_{2}\left[\sum_{v\in V_{G_{n-1}}}d\left(v\right)r\left(u_{n-1},v\right)+\left(1+\frac{2\cdot (2m-2)}{2m}\right)\left(\left(4m+2\right)n-1\right)+\left(\frac{4m^{2}-1}{3}+\frac{2\cdot (2m-2)}{2m}\right)\right]\\ \; & +p_{3}\left[\sum_{v\in V_{G_{n-1}}}d\left(v\right)r\left(u_{n-1},v\right)+\left(1+\frac{3\cdot (2m-3)}{2m}\right)\left(\left(4m+2\right)n-1\right)+\left(\frac{4m^{2}-1}{3}+\frac{3\cdot (2m-3)}{2m}\right)\right]\\ \; & +\cdots \\ \; & +p_{m-3}[\sum_{v\in V_{G_{n-1}}}d\left(v\right)r\left(u_{n-1},v\right)+\left(1+\frac{\left(m-3)\cdot (m+3\right)}{2m}\left(\left(4m+2\right)n-1\right)+\left(\frac{4m^{2}-1}{3}+\frac{\left(m-3)\cdot (m+3\right)}{2m}\right)\right]\\ \; & +p_{m-2}[\sum_{v\in V_{G_{n-1}}}d\left(v\right)r\left(u_{n-1},v\right)+\left(1+\frac{\left(m-2)\cdot (m+2\right)}{2m}\left(\left(4m+2\right)n-1\right)+\left(\frac{4m^{2}-1}{3}+\frac{\left(m-2)\cdot (m+2\right)}{2m}\right)\right]\\ & +p_{m-1}[\sum_{v\in V_{G_{n-1}}}d\left(v\right)r\left(u_{n-1},v\right)+\left(1+\frac{\left(m-1)\cdot (m+1\right)}{2m}\left(\left(4m+2\right)n-1\right)+\left(\frac{4m^{2}-1}{3}+\frac{\left(m-1)\cdot (m+1\right)}{2m}\right)\right]\\ \; & +\left(1-p_{1}-p_{2}-\cdots -p_{m-1}\right)\left[\sum_{v\in V_{G_{n-1}}}d\left(v\right)r\left(u_{n-1},v\right)+\left(1+\frac{m\cdot m}{2m}\right)\left(\left(4m+2\right)n-1\right)+\left(\frac{4m^{2}-1}{3}+\frac{m\cdot m}{2m}\right)\right]. \end{array}$$

Substitute the expectation for the above equation, $E\left(R_{n}\right)=R_{n}$, we obtain

$$\begin{array}{cc} R_{n}= & R_{n-1}+\left\{\left(2m^{2}+5m+2\right)-\sum_{i=1}^{m-1}\left[\left(2m^{2}+5m+2\right)-\frac{2m+i\cdot (2m-i)}{m}\left(2m+1\right)\right]p_{i}\right\}n\\ \; & +\sum_{i=1}^{m-1}\left[\left(2m^{2}+5m+2\right)-\frac{2m+i\cdot (2m-i)}{m}\left(2m+1\right)\right]p_{i}-\frac{2m^{2}+15m+10}{3}. \end{array}$$

Let

$$V=\sum_{i=1}^{m-1}\left[\left(2m^{2}+5m+2\right)-\frac{2m+i\cdot (2m-i)}{m}\left(2m+1\right)\right]p_{i}.$$

$$W_{i}=\left(2m+1\right)\left[\left(2m^{2}+5m+2\right)-\frac{2m+i\cdot (2m-i)}{m}\left(2m+1\right)\right].$$

Hence,

$$R_{n}=R_{n-1}+\left[\left(2m^{2}+5m+2\right)-V\right]n+V-\frac{2m^{2}+15m+10}{3}.$$

By the calculation

$$R_{1}=E\left(\sum_{v\in V_{G_{n}}}d\left(v\right)r\left(u_{1},v\right)\right)=\frac{4m^{2}-1}{3}.$$

Based on the above results, we have

$$\begin{array}{cc} R_{n}= & \left\{\frac{\left(2m^{2}+5m+2\right)}{2}-\frac{1}{2}\sum_{i=1}^{m-1}\left[\left(2m^{2}+5m+2\right)-\frac{2m+i\cdot (2m-i)}{m}\left(2m+1\right)\right]p_{i}\right\}n^{2}\\ \; & +\{\frac{1}{2}\sum_{i=1}^{m-1}\left[\left(2m^{2}+5m+2\right)-\frac{2m+i\cdot (2m-i)}{m}\left(2m+1\right)\right]p_{i}+\frac{2m^{2}-15m-14}{6}\}n+1. \end{array}$$

Thus,

$$\begin{array}{c} R_{n}=\left[\frac{\left(2m^{2}+5m+2\right)}{2}-\frac{1}{2}V\right]n^{2}+\left[\frac{1}{2}V+\frac{2m^{2}-15m-14}{6}\right]n+1. \end{array}$$

Substituting $R_{n}$ into (6), we have

$$\begin{array}{cc} E(Kf^{*}\left(G_{n+1}\right))= & E\left(Kf^{*}\left(G_{n}\right)\right)+\left(4m+2\right)R_{n}+\frac{16m^{3}+56m^{2}+32m+4}{3}n+\frac{8m^{3}-14m-3}{3}\\ = & E\left(Kf^{*}\left(G_{n}\right)\right)+\left(4m+2\right)\left\{\left[\frac{\left(2m^{2}+5m+2\right)}{2}-\frac{1}{2}V\right]n^{2}+\left[\frac{1}{2}V+\frac{2m^{2}-15m-14}{6}\right]n+1\right\}\\ \; & +\frac{16m^{3}+56m^{2}+32m+4}{3}n+\frac{8m^{3}-14m-3}{3}. \end{array}$$

By these calculations, $E\left(Kf^{*}\left(G_{1}\right)\right)=\frac{8m^{3}-2m}{3}$.

Finally, we obtain the expected value formula

$$\begin{array}{cc} E(Kf^{*}\left(G_{n}\right) & )=\left\{\left(4m^{3}+12m^{2}+9m+2\right)-\left(2m+1\right)\sum_{i=1}^{m-1}\left[\left(2m^{2}+5m+2\right)-\frac{2m+i(2m-i)}{m}\left(2m+1\right)\right]p_{i}\right\}\frac{n^{3}}{3}\\ \; & +\left\{\frac{4m^{3}-4m^{2}-19m-8}{3}+\left(2m+1\right)\sum_{i=1}^{m-1}\left[\left(2m^{2}+5m+2\right)-\frac{2m+i(2m-i)}{m}\left(2m+1\right)\right]p_{i}\right\}n^{2}\\ \; & -\left\{\left(8m^{2}-8m-9\right)+2\left(2m+1\right)\sum_{i=1}^{m-1}\left[\left(2m^{2}+5m+2\right)-\frac{2m+i(2m-i)}{m}\left(2m+1\right)\right]p_{i}\right\}\frac{n}{3}-1. \end{array}$$

Thus,

$$\begin{array}{cc} E(Kf^{*}\left(G_{n}\right))= & \left[\left(4m^{3}+12m^{2}+9m+2\right)-\left(2m+1\right)V\right]\frac{n^{3}}{3}+\left[\frac{4m^{3}-4m^{2}-19m-8}{3}+\left(2m+1\right)V\right]n^{2}\\ \; & -\left[\left(8m^{2}-8m-9\right)+2\left(2m+1\right)V\right]\frac{n}{3}-1. \end{array}$$

as desired.

In particular, if we let $\left(p_{1},p_{2},p_{3},\ldots ,p_{m-1},p_{m}\right)$ = (1,0,0,…,0,0), (0,1,0,…,0,0), (0,0,1,…,0,0), …, (0,0,0,…,1,0), (0,0,0,…,0,1) or (0,0,0,…,0,0), by Theorem 1, we can obtain the multiplicative degree-Kirchhoff index of the polygonal meta-chain $M_{n}\left(p_{1}=1\right)$,the polygonal ortho-chain $O_{n}^{1}$, $O_{n}^{2}$, $O_{n}^{3}$, …,$O_{n}^{m-2}\left(p_{i+1}=1,1\leq i\leq m-2\right)$, the polygonal para-chain $L_{n}\left(p_{m}=1\right)$, as

$$\begin{array}{cc} \; & Kf^{*}\left(M_{n}\right)=\frac{16m^{3}+12m^{2}-1}{3m}n^{3}+\frac{16m^{4}-16m^{3}-28m^{2}-2m+3}{3m}n^{2}-\frac{8m^{4}-14m^{2}-5m+2}{3m}n-1,\\ \; & Kf^{*}\left(O_{n}^{1}\right)=\frac{24m^{3}+8m^{2}-10m-4}{3m}n^{3}+\frac{16m^{4}-40m^{3}-16m^{2}+28m+12}{3m}n^{2}-\frac{8m^{4}-16m^{3}-6m^{2}+15m+8}{3m}n-1,\\ \; & Kf^{*}\left(O_{n}^{2}\right)=\frac{32m^{3}-4m^{2}-28m-9}{3m}n^{3}+\frac{16m^{4}-64m^{3}+20m^{2}+82m+27}{3m}n^{2}-\frac{8m^{4}-32m^{3}+18m^{2}+51m+18}{3m}n-1,\\ \; & \;\;\;\;\vdots \;\;\;\;\;\;\;\;\;\;\;\;\;\;\;\;\;\;\;\;\;\;\vdots \;\;\;\;\;\;\;\;\;\;\;\;\;\;\;\;\;\;\;\;\;\;\vdots \;\;\;\;\;\\ \; & Kf^{*}\left(O_{n}^{m-3}\right)=\frac{4m^{4}+12m^{3}-7m^{2}-14m-4}{3m}n^{3}+\frac{4m^{4}-4m^{3}+29m^{2}+40m+12}{3m}n^{2}-\frac{8m^{3}+24m^{2}+23m+8}{3m}n-1,\\ \; & Kf^{*}\left(O_{n}^{m-2}\right)=\frac{4m^{4}+12m^{3}+5m^{2}-2m-1}{3m}n^{3}+\frac{4m^{4}-4m^{3}-7m^{2}+4m+3}{3m}n^{2}-\frac{8m^{3}-m+2}{3m}n-1,\\ \; & Kf^{*}\left(L_{n}\right)=\frac{4m^{4}+12m^{3}+9m^{2}+2m}{3m}n^{3}+\frac{4m^{4}-4m^{3}-19m^{2}-8m}{3m}n^{2}-\frac{8m^{3}-8m^{2}-9m}{3m}n-1. \end{array}$$

$$Kf^{*}\left(O_{n}^{i}\right)=\left[\left(4m^{3}+12m^{2}+9m+2\right)-W_{i+1}\right]\frac{n^{3}}{3}+\left[\frac{4m^{3}-4m^{2}-19m-8}{3}+W_{i+1}\right]n^{2}-\left[\left(8m^{2}-8m-9\right)+2W_{i+1}\right]\frac{n}{3}-1,$$

Obviously,

$$Kf^{*}\left(M_{n}\right)+Kf^{*}\left(L_{n}\right)=Kf^{*}\left(O_{n}^{1}\right)+Kf^{*}\left(O_{n}^{2}\right)+\cdots +Kf^{*}\left(O_{n}^{m-2}\right).$$

**Corollary** **1.** For a random polygonal chain $G_{n}\;\left(n\geq 3\right)$, the para-chain $L_{n}$ reaches the maximum and the meta-chain $M_{n}$ reaches the minimum of $E(kf^{*}\left(G_{n}\right))$.

**Proof.**  Though Theorem 1, we have

$$\begin{array}{cc} E(Kf^{*}\left(G_{n}\right)) & =\sum_{i=1}^{m-1}\left(-W_{i}\frac{n^{3}}{3}+W_{i}n^{2}-2W_{i}\frac{n}{3}\right)p_{i}+\left(4m^{3}+12m^{2}+9m+2\right)\frac{n^{3}}{3}\\ + & \frac{4m^{3}-4m^{2}-19m-8}{3}n^{2}-\frac{8m^{2}-8m-9}{3}n-1. \end{array}$$

When n≥3, by taking the partial derivative of $E(Kf^{*}\left(G_{n}\right))$, one has

$$\begin{array}{cc} \; & \frac{\partial E(Kf^{*}\left(G_{n}\right))}{\partial p_{i}}=-W_{i}\frac{n^{3}}{3}+W_{i}n^{2}-\frac{2}{3}W_{i}n<0.\\ \; & \frac{\partial E(Kf^{*}\left(G_{n}\right))}{\partial p_{1}}=-\frac{4m^{4}-4m^{3}-3m^{2}+2m+1}{m}\frac{n^{3}}{3}+\frac{4m^{4}-4m^{3}-3m^{2}+2m+1}{m}n^{2}\\ \; & \;\;\;\;\;\;\;\;\;\;\;\;\;\;\;\;\;\;\;\;\;\;\;\;\;-\frac{2}{3}\cdot \frac{4m^{4}-4m^{3}-3m^{2}+2m+1}{m}n<0,\\ \; & \frac{\partial E(Kf^{*}\left(G_{n}\right))}{\partial p_{2}}=-\frac{4m^{4}-12m^{3}+m^{2}+12m+4}{m}\frac{n^{3}}{3}+\frac{4m^{4}-12m^{3}+m^{2}+12m+4}{m}n^{2}\\ \; & \;\;\;\;\;\;\;\;\;\;\;\;\;\;\;\;\;\;\;\;\;\;\;\;\;-\frac{2}{3}\cdot \frac{4m^{4}-12m^{3}+m^{2}+12m+4}{m}n<0,\\ \; & \frac{\partial E(Kf^{*}\left(G_{n}\right))}{\partial p_{3}}=-\frac{4m^{4}-20m^{3}+13m^{2}+30m+9}{m}\frac{n^{3}}{3}+\frac{4m^{4}-20m^{3}+13m^{2}+30m+9}{m}n^{2}\\ \; & \;\;\;\;\;\;\;\;\;\;\;\;\;\;\;\;\;\;\;\;\;\;\;\;\;-\frac{2}{3}\cdot \frac{4m^{4}-20m^{3}+13m^{2}+30m+9}{m}n<0,\\ \; & \;\;\;\;\;\;\;\;\vdots \;\;\;\;\;\;\;\;\;\;\;\;\;\;\;\;\;\;\;\;\;\;\;\vdots \;\;\;\;\;\;\;\;\;\;\;\;\;\;\;\;\;\;\;\;\;\vdots \;\;\;\;\;\;\;\;\;\\ \; & \frac{\partial E(Kf^{*}\left(G_{n}\right))}{\partial p_{m-1}}=-\frac{4m^{2}+4m+1}{m}\frac{n^{3}}{3}+\frac{4m^{2}+4m+1}{m}n^{2}-\frac{2}{3}\cdot \frac{4m^{2}+4m+1}{m}n<0. \end{array}$$

When $\left(p_{1},p_{2},p_{3},\ldots ,p_{m-1},p_{m}\right)$=(0,0,0,…,0,1)(i.e., $p_{m}=1$), the para-chain $L_{n}$ reaches the maximum of $E(Kf^{*}\left(G_{n}\right))$, (i.e., $G_{n}≅L_{n}$). If $p_{1}+p_{2}+p_{3}+\ldots +p_{m-1}=1$, let $p_{m-1}=1-p_{1}-p_{2}-\ldots -p_{m-2}\;\left(0\leq p_{1}\leq 1,\;0\leq p_{2}\leq 1,\ldots ,0\leq p_{m-2}\leq 1\right)$, Then

$$\begin{array}{cc} E(Kf^{*}\left(G_{n}\right))= & \sum_{i=1}^{m-2}\left(-W_{i}\frac{n^{3}}{3}+W_{i}n^{2}-2W_{i}\frac{n}{3}\right)p_{i}+\left(-W_{m-1}\frac{n^{3}}{3}+W_{m-1}n^{2}-2W_{m-1}\frac{n}{3}\right)\left(1-p_{1}-p_{2}-\cdots -p_{m-2}\right)\\ \; & +\left(4m^{3}+12m^{2}+9m+2\right)\frac{n^{3}}{3}+\frac{4m^{4}-4m^{2}-19m-8}{3}n^{2}-\frac{8m^{2}-8m-9}{3}n-1. \end{array}$$

Therefore,

$$\begin{array}{cc} \; & \frac{\partial E(Kf^{*}\left(G_{n}\right))}{\partial p_{i}}=-\left(W_{i}-W_{m-1}\right)\frac{n^{3}}{3}+\left(W_{i}-W_{m-1}\right)n^{2}-\frac{2}{3}\left(W_{i}-W_{m-1}\right)n<0.\\ \; & \frac{\partial E(Kf^{*}\left(G_{n}\right))}{\partial p_{1}}=-\left(4m^{3}-4m^{2}-7m-2\right)\frac{n^{3}}{3}+\left(4m^{3}-4m^{2}-7m-2\right)n^{2}-\frac{2}{3}\cdot \left(4m^{3}-4m^{2}-7m-2\right)n<0,\\ \; & \frac{\partial E(Kf^{*}\left(G_{n}\right))}{\partial p_{2}}=-\frac{4m^{4}-12m^{3}-3m^{2}+8m+3}{m}\frac{n^{3}}{3}+\frac{4m^{4}-12m^{3}-3m^{2}+8m+3}{m}n^{2}\\ \; & \;\;\;\;\;\;\;\;\;\;\;\;\;\;\;\;\;\;\;\;\;\;\;\;\;-\frac{2}{3}\cdot \frac{4m^{4}-12m^{3}-3m^{2}+8m+3}{m}n<0,\\ \; & \;\;\;\;\;\;\;\;\vdots \;\;\;\;\;\;\;\;\;\;\;\;\;\;\;\;\;\;\;\;\;\;\;\vdots \;\;\;\;\;\;\;\;\;\;\;\;\;\;\;\;\;\;\;\;\;\vdots \;\;\;\;\;\;\;\;\;\\ \; & \frac{\partial E(Kf^{*}\left(G_{n}\right))}{\partial p_{m-2}}=-\frac{12m^{2}+12m+3}{m}\frac{n^{3}}{3}+\frac{12m^{2}+12m+3}{m}n^{2}-\frac{2}{3}\cdot \frac{12m^{2}+12m+3}{m}n<0. \end{array}$$

Thus, $\left(p_{1},p_{2},p_{3},\ldots ,p_{m-1},p_{m}\right)$=(0,0,0,…,1,0)(i.e., $p_{m-1}=1$), $E(Kf^{*}\left(G_{n}\right))$ cannot be minimized. As above, If $p_{1}+p_{2}+p_{3}+\ldots +p_{i}=1$, let $p_{i}=1-p_{1}-p_{2}-\ldots -p_{i-1}\;\left(0\leq p_{1}\leq 1,\;0\leq p_{2}\leq 1,\ldots ,0\leq p_{i-1}\leq 1\right)$, (i≥3); then, we have

$$\begin{array}{cc} E(Kf^{*}\left(G_{n}\right))= & \sum_{i=1}^{m-3}\left(-W_{i}\frac{n^{3}}{3}+W_{i}n^{2}-2W_{i}\frac{n}{3}\right)p_{i}+\left(-W_{m-2}\frac{n^{3}}{3}+W_{m-2}n^{2}-2W_{m-2}\frac{n}{3}\right)\left(1-p_{1}-p_{2}-\cdots -p_{m-3}\right)\\ \; & +\left(4m^{3}+12m^{2}+9m+2\right)\frac{n^{3}}{3}+\frac{4m^{3}-4m^{2}-19m-8}{3}n^{2}-\frac{8m^{2}-8m-9}{3}n-1. \end{array}$$

Therefore,

$$\begin{array}{cc} \; & \frac{\partial E(Kf^{*}\left(G_{n}\right))}{\partial p_{i}}=-\left(W_{i}-W_{m-2}\right)\frac{n^{3}}{3}+\left(W_{i}-W_{m-2}\right)n^{2}-\frac{2}{3}\left(W_{i}-W_{m-2}\right)n<0,\left(m-3\geq 3\right). \end{array}$$

The minimum value can only be reached if $p_{1}+p_{2}=1$. Then let $p_{1}=1-p_{2}\;\left(0\leq p_{2}\leq 1\right)$

$$\begin{array}{cc} E(Kf^{*}\left(G_{n}\right))= & \left(-W_{1}\frac{n^{3}}{3}+W_{1}n^{2}-2W_{1}\frac{n}{3}\right)\left(1-p_{2}\right)+\left(-W_{2}\frac{n^{3}}{3}+W_{2}n^{2}-2W_{2}\frac{n}{3}\right)p_{2}\\ \; & +\left(4m^{3}+12m^{2}+9m+2\right)\frac{n^{3}}{3}+\frac{4m^{3}-4m^{2}-19m-8}{3}n^{2}-\frac{8m^{2}-8m-9}{3}n-1. \end{array}$$

Thus,

$$\frac{\partial E(G\left(Kf_{n}^{*}\right))}{\partial p_{2}}=\left(W_{1}-W_{2}\right)\frac{n^{3}}{3}-\left(W_{1}-W_{2}\right)n^{2}+\frac{2}{3}\left(W_{1}-W_{2}\right)n>0.$$

Therefore, $E(Kf^{*}\left(G_{n}\right))$ reaches its minimum value when $p_{2}=0$ (i.e., $\;p_{1}=1)$; that is $G_{n}≅M_{n}$.

□

## 3. The Average Values for the Multiplicative Degree-Kirchhoff Index

Let $\Theta_{n}$ be the set of all polygonal chains with *n* polygons. Here, we calculate the average value of the multiplicative degree-Kirchhoff index.

$$Kf_{avr}^{*}\left(\Theta_{n}\right)=\frac{1}{|\Theta_{n}|}\sum_{G\in \Theta_{n}}Kf^{*}\left(G\right).$$

In order to obtain the average value $Kf_{avr}^{*}\left(\Theta_{n}\right)$, we let $p_{1}=p_{2}=\ldots =p_{m}=\frac{1}{m}$ in the random polygonal chain of $E(Kf^{*}\left(G_{n}\right))$. According to Theorem 1, we have

**Theorem** **2.** The $Kf_{avr}^{*}\left(\Theta_{n}\right)$(n≥1) for the multiplicative degree-Kirchhoff index of the random chain $G_{n}$ is

$$\begin{array}{cc} E(Kf^{*}\left(G_{n}\right))= & \left[\left(4m^{3}+12m^{2}+9m+2\right)-\frac{1}{k}\sum_{i=1}^{m-1}W_{i}\right]\frac{n^{3}}{3}+\left[\frac{4m^{3}-4m^{2}-19m-8}{3}+\frac{1}{m}\sum_{i=1}^{m-1}W_{i}\right]n^{2}\\ \; & -\left[\left(8m^{2}-8m-9\right)+\frac{2}{m}\sum_{i=1}^{m-1}W_{i}\right]\frac{n}{3}-1. \end{array}$$

After calculation, we obtain the equations

$$\begin{array}{cc} Kf_{avr}^{*}\left(\Theta_{n}\right) & =\frac{1}{m}Kf^{*}\left(M_{n}\right)+\frac{1}{m}Kf^{*}\left(O_{n}^{1}\right)+\frac{1}{m}Kf^{*}\left(O_{n}^{2}\right)+\cdots +\frac{1}{m}Kf^{*}\left(O_{n}^{m-2}\right)+\frac{1}{m}Kf^{*}\left(L_{n}\right). \end{array}$$

## 4. Concluding Remarks

In this paper, we compute an expression for the expected value of the multiplicative degree-Kirchhoff index of a random polygonal chain. We also calculate the extremal value and average value of this index. Polygonal chemicals have various molecular structures, and their physicochemical properties are becoming increasingly important; refer to [33,34,35]. These studies have important applications for us to solve some chemical problems related to life and production, as well as for us to predict the physical and chemical properties of molecules and synthesize new compounds and new drugs.

Nowadays, computational chemists can identify the various physical, chemical and pharmaceutical properties of molecules by statistical methods using a large amount of data. Topological indices based on the distance between vertices of graphs play an important role in characterizing molecular graphs and establishing the relationship between molecular structures and features and are used to predict the physicochemical properties and biological activities of compounds. With the rapid development of science and technology, the demand for new materials and drugs in the manufacturing and pharmaceutical fields is increasing day by day. In order to purposefully and quickly synthesize new substances, the topological index has once again become a research hotspot [36,37].
